# 

**Published:** 1895-03-02

**Authors:** 


### CONTENTS.

**The Ethical in Modern Medicine** - - - - - - 377

**Neuritis and Neuralgia** - - - - - - - - 378

**The Study of Man** - - - - - - - - - 379

**Nervous Diseases and Modern Life** - - - - - 380

**Annotations.**—Infectious Diseases: The Fruits of Notification—John Hunter as a Biologist—Influenza: Do Doctors Know Anything About It? - - - - - - - - - 381

**Notes and News** - - - - - - - - - 382

**Editor's Letter Fox** - - - - - - - - - 386

**Medical Progress and Hospital Clinics:**

On Some Minor Anal and Rectal Ailments—II. - - - - 383

Progress in Medicine.—Diabetes - - - - - - - 384

Therapeutic Notes - - - - - - - - - 385

**The Institutional Workshop:**

Hospital Administration. — Paying Patients in General Hospitals - - - - - - - - - 387

The Pay System.—The Great Northern Central Hospital - 389

Hospital Festivals and Meetings - - - - - - 391

### EXTRA SUPPLEMENT.—THE NURSING MIRROR.

**News from the Nursing World.**—Lectures at St. Mary's—Lewisham Infirmary—Dr. James Anderson—Nurse and Caretaker—Private Nurses at Leeds—Woolton—An Excellent Example—Good Management—Nurses at Northampton—A Homœopathic Hospital—A Girl Nurse—The President and the Hospitals—Italy in ay—Nurses at Vancouver—A Parisian Maternity Charity—Short Items - - - - - - clxi

**Elementary Anatomy and Surgery for Nurses.**—By W. McAdam Eccles, M.B., M.S., F.R.C.S. - - - - - clxi

**Appointments** - - - - - - - - - clxii

**Nur ing in New South Wales** - - - - - - clxii

**Where to Go** - - - - - - - - - clxii

**Troubles at Lewisham** - - - - - - - clxiii

**Burdett's Official Nursing Directory** - - - - clxiii

**Death in Our Ranks** - - - - - - - - clxiii

**Minor Appointments** - - - - - - - - clxiii

**Everybody's Opinion** - - - - - - - - c xiv

**Notes and Queries—Wants and Workers** - - - - clxiv

